# Serum anti‑KIAA0513 antibody as a common biomarker for mortal atherosclerotic and cancerous diseases

**DOI:** 10.3892/mi.2024.169

**Published:** 2024-06-19

**Authors:** Takaki Hiwasa, Yoichi Yoshida, Masaaki Kubota, Shu-Yang Li, Bo-Shi Zhang, Tomoo Matsutani, Seiichiro Mine, Toshio Machida, Masaaki Ito, Satoshi Yajima, Mikako Shirouzu, Shigeyuki Yokoyama, Mizuki Sata, Kazumasa Yamagishi, Hiroyasu Iso, Norie Sawada, Shoichiro Tsugane, Minoru Takemoto, Aiko Hayashi, Koutaro Yokote, Yoshio Kobayashi, Kazuyuki Matsushita, Koichiro Tatsumi, Hirotaka Takizawa, Go Tomiyoshi, Hideaki Shimada, Yoshinori Higuchi

**Affiliations:** 1 Department of Neurological Surgery, Graduate School of Medicine, Chiba University, Chiba 260-8670, Japan; 2 Department of Biochemistry and Genetics, Graduate School of Medicine, Chiba University, Chiba 260-8670, Japan; 3 Department of Clinical Oncology, Toho University Graduate School of Medicine, Tokyo 143-8541, Japan; 4 Department of Rehabilitation in Traditional Chinese Medicine, The Second Affiliated Hospital of Zhejiang University School of Medicine, Hangzhou, Zhejiang 310003, P.R. China; 5 Department of Neurological Surgery, Chiba Prefectural Sawara Hospital, Chiba 287-0003, Japan; 6 Department of Neurological Surgery, Chiba Cerebral and Cardiovascular Center, Chiba 290-0512, Japan; 7 Department of Neurosurgery, Eastern Chiba Medical Center, Chiba 283-8686, Japan; 8 Department of Gastroenterological Surgery and Clinical Oncology, Toho University Graduate School of Medicine, Tokyo 143-8541, Japan; 9 Laboratory for Protein Functional and Structural Biology, RIKEN Center for Biosystems Dynamics Research, Yokohama, Kanagawa 230-0045, Japan; 10 RIKEN Structural Biology Laboratory, Yokohama, Kanagawa 230-0045, Japan; 11 Department of Public Health Medicine, Faculty of Medicine, and Health Services Research and Development Center, University of Tsukuba, Tsukuba 305-8575, Japan; 12 Department of Preventive Medicine and Public Health, Keio University School of Medicine, Tokyo 160-8582, Japan; 13 Public Health, Department of Social Medicine, Osaka University Graduate School of Medicine, Suita, Osaka 565-0871, Japan; 14 Epidemiology and Prevention Group, Center for Public Health Sciences, National Cancer Center, Tokyo 104-0045, Japan; 15 Department of Endocrinology, Hematology and Gerontology, Graduate School of Medicine, Chiba University, Chiba 260-8670, Japan; 16 Department of Diabetes, Metabolism and Endocrinology, School of Medicine, International University of Health and Welfare, Chiba 286-8686, Japan; 17 Department of Cardiovascular Medicine, Graduate School of Medicine, Chiba University, Chiba 260-8670, Japan; 18 Department of Laboratory Medicine & Division of Clinical Genetics, Chiba University Hospital, Chiba 260-8677, Japan; 19 Department of Respirology, Graduate School of Medicine, Chiba University, Chiba 260-8670, Japan; 20 Port Square Kashiwado Clinic, Kashiwado Memorial Foundation, Chiba 260-0025, Japan; 21 Medical Project Division, Research Development Center, Fujikura Kasei Co., Saitama 340-0203, Japan

**Keywords:** atherosclerosis, acute ischemic stroke, diabetes mellitus, cardiovascular disease, solid cancer, antibody biomarker

## Abstract

Numerous antibody biomarkers have been reported for cancer and atherosclerosis-related diseases. The major complications of atherosclerosis and diabetes mellitus (DM) are acute ischemic stroke (AIS), cardiovascular disease (CVD) and chronic kidney disease (CKD). Cancer development is accompanied by arterial disorders, such as angiogenesis and atherosclerosis, and DM is a risk factor for the development of certain types of cancer. Atherosclerosis-related diseases and cancers are therefore interrelated and could be detected using a common biomarker. In the present study, the initial screening using the protein array method identified KIAA0513 as an antigen recognized by serum IgG antibodies in patients with atherosclerosis. The amplified luminescent proximity homogeneous assay-linked immunosorbent assay revealed significantly higher serum antibody levels against recombinant KIAA0513 protein in patients with AIS, transient ischemic attack (TIA), DM, CVD, obstructive sleep apnea syndrome (OSAS), CKD and solid cancers, such as esophageal, gastric, colon, lung and breast cancers, compared with healthy donors. A receiver operating characteristic (ROC) analysis revealed that the highest areas under the ROC curves of anti-KIAA0513 antibodies were obtained for esophageal cancer, nephrosclerosis-type CKD and DM. Spearman's correlation analysis revealed that serum anti-KIAA0513 antibody levels were associated with maximum intima-media thickness and plaque score, which are indices of atherosclerosis and stenosis. Serum anti-KIAA0513 antibody markers appear to be useful for diagnosing AIS, TIA, DM, CVD, OSAS, CKD and solid cancers, and may reflect common arterial alterations leading to atherosclerotic and cancerous diseases.

## Introduction

In recent years, various disease biomarkers have been discovered, and the development of simple blood tests is underway to determine the pathological condition, predict the onset of disease and its prognosis, and identify preventive/therapeutic targets. In terms of biomarker species, studies have reported enzyme, antigen, and, in recent years, nucleotide markers (1-3). However, there are still some reports on antibody markers, which include heat-shock 60-kD protein 1(4), replication protein A2(5), programmed cell death 11(6), metalloproteinase 1, chromobox homolog 1, chromobox homolog 5(7), DnaJ heat shock protein family (Hsp40) member C2(8), adaptor-related protein complex 3 subunit delta 1(9), serpin peptidase inhibitor, clade E member 1(10), death-inducer obliterator 1, cleavage and polyadenylation specificity factor 2, forkhead box J2(11) and thiosulfate sulfurtransferase-like domain-containing 2(12) for acute ischemic stroke (AIS); ATPase, Ca^++^ transporting, plasma membrane 4(10), bone morphogenetic protein 1 (3,13), deoxyhypusine synthase (14), SH3 domain-binding protein 5(15), prolyl carboxypeptidase (16), low-density lipoprotein receptor-related protein-associated protein 1(17) and additional sex combs-like 2(18) for atherosclerosis; nardilysin (19) for acute cardiac syndrome; and insulin (20), glutamic acid decarboxylase (21), adiponectin (22) and growth arrest and DNA-damage-inducible gene 34 (23,24), and proprotein convertase subtilisin/kexin type 9(25) for diabetes mellitus (DM).

The anti-p53 antibody is a typical antibody biomarker for cancer that has been used in clinical practical for diagnosing, monitoring and predicting the prognosis of esophageal cancer (EC) and head and neck cancer (26,27). Further application of the serological identification of antigens by cDNA expression cloning and the protein array method have identified autoantibodies against tumor-associated calcium signal transducer 2(28), solute carrier family 2/facilitated glucose transporter, member 1(29), tripartite motif-containing 21(30), myomegalin (31), makorin 1(32), esophageal carcinoma SEREX antigen (33), cyclin L2(34), cofilin, β-actin (35) and WD repeat-containing protein 1(36) for EC; FBP-interacting repressor for colorectal cancer (CRC) (37) and gastric cancer (GC) (38); SH3 domain, GRB2-like 1(39) and filamin C (40) for glioma; EP300-interacting inhibitor of differentiation 3 for non-functional pancreatic neuroendocrine tumors (41); wingless-type MMTV integration site family, member 7 for biliary cancer (42); and coatomer protein complex subunit epsilon (43), differential screening-selected gene aberrant in neuroblastoma (44), and sorting nexins 16(45) for obstructive sleep apnea syndrome (OSAS). Here, we report on serum antibodies against KIAA0513 (s-KIAA0513-Ab) as a broad-spectrum biomarker applicable to atherosclerosis-related diseases such as ischemic stroke, cardiovascular disease (CVD), chronic kidney disease (CKD), DM and solid cancers.

## Materials and methods

### Patients and control sera

The present study was conducted according to the guidelines of the Declaration of Helsinki and approved by the Local Ethical Review Board of the Chiba University, Graduate School of Medicine (Chiba, Japan), as well as by the review boards of the participating hospitals (approval no. 2018-320). The Ethics Committee of Toho University, Graduate School of Medicine, Tokyo, Japan (No. A18103_A17052_A16035_A16001_26095_25024_24038_22047_22047) and Port Square Kashiwado Clinic, Kashiwado Memorial Foundation, China, Japan (approval no. 2012-001) also approved the study protocol. Sera were collected from patients who had provided written informed consent. Each serum sample was centrifuged at 3,000 x g for 10 min at 4˚C, and the supernatant was stored at -80˚C until use.

Serum samples collected from patients with AIS, transient ischemic attack (TIA), asymptomatic ischemic stroke (Asympt-CI), chronic-phase cerebral infarction (cCI), and deep and subcortical white matter hyperintensity (DSWMH) were obtained from Chiba Prefectural Sawara Hospital. The stroke subtypes were determined according to the criteria of the Trial of Org 10172 in the Acute Stroke Treatment classification system (46), and large-artery atherosclerosis and small-artery occlusion (lacune) were included as AIS and ischemic stroke. Samples from patients with DM, CVD and OSAS were obtained from the Chiba University Hospital. CVD included acute myocardial infarction (AMI) and unstable angina. The serum samples of patients with AIS, TIA and AMI were obtained within 2 weeks following disease onset. Samples collected from patients with CKD were obtained from the Kumamoto cohort (47,48), whereas those collected from patients with EC, GC, CRC, lung cancer (LC), and breast cancer (BC) were obtained from the Department of Surgery, Toho University Hospital. Serum samples from healthy donors (HDs) were obtained from Chiba University, Port Square Kashiwado Clinic, the National Hospital Organization, Shimoshizu Hospital (Yotsukaido, Japan), and Chiba Prefectural Sawara Hospital (Katori, Japan). For comparisons with TIA and AIS, serum samples from HDs were selected from patients who exhibited no abnormalities in cranial magnetic resonance imaging.

### ProtoArray^®^ screening

The initial screening was performed using ProtoArray^®^ Human Protein Microarrays v4.0 (Thermo Fisher Scientific, Inc.), which were loaded with 9,480 species of proteins, as previously described (11,14,15). In total, 20 serum samples (10 each from the patients and HDs) were employed to detect antigens specifically recognized by IgG antibodies in the patient sera. The complete results from the ProtoArray^®^ screening are presented in Table SI.

### Expression and purification of KIAA0513 protein

A sequence encoding cDNA of the isoform c of human KIAA0513 (accession no. NP_001284695.1) was cloned into pGEX-6P (Cytiva). The expression of the cDNA product was induced by treating *Escherichia coli* (*E. coli*) KRX (Promega Corp.) cells harboring the pGEX-6P-KIAA0513 and pMINOR with 0.5 mM isopropyl-β-D-thiogalactoside (FUJIFILM Wako Pure Chemical Corporation) at 37˚C for 3 h (49). The cells were lysed by sonication in phosphate-buffered saline (FUJIFILM Wako Pure Chemical Corporation) containing 1% Triton X-100 (FUJIFILM Wako Pure Chemical Corporation) and 2 mM dithiothreitol (FUJIFILM Wako Pure Chemical Corporation). The glutathione S-transferase (GST)-fused proteins were specifically bound to Glutathione-Sepharose 4 Fast Flow medium (Cytiva) and HiPrep 26/10 Desalting column (1 ml) (Cytiva), followed by washing with 30 ml of phosphate-buffered saline. GST-KIAA0513 protein was eluted with 10 mM glutathione (Wako Pure Chemicals) and 2 mM dithiothreitol in phosphate-buffered saline, and concentrated to 3.5 mg/ml in phosphate-buffered saline containing 2 mM dithiothreitol as previously described (49). Protein concentration was determined by Bradford Protein Assay (Bio-Rad Laboratories, Inc.).

### Western blot analysis

Purified GST-KIAA0513 and the control GST proteins (0.3 µg/lane) were electrophoresed through sodium dodecyl sulfate-polyacrylamide (11%) gels, followed by directly staining with 0.05% Coomassie Brilliant Blue (Nacalai Tesque) in 50% methanol and 10% acetic acid for 1 h at 25˚C, or blotting onto nitrocellulose membranes (S045A330R, Advantec). The membranes were blocked with 0.1% dry milk (Megmilk Snow Brand Co., Ltd.) in 150 mM NaCl, 20 mM Tris-HCl (pH 7.6) and 0.1% Tween-20 (TBS-T) at 25˚C for 1 h, and then treated with anti-GST (goat, ab6613, Abcam), anti-KIAA0513 (rabbit, HPA012866, Atlas Antibodies) antibodies at a final concentration of 0.2 µg/ml, or the patient sera (1/1,000 fold dilution) at 25˚C overnight. The membranes were washed five times with TBS-T and treated with HRP-conjugated secondary antibodies (HRP-conjugated donkey anti-goat IgG, sc-2020, Santa Cruz Biotechnology, Inc., 1/30,000-fold dilution; HRP-conjugated goat anti-rabbit IgG, 111-035-003, Jackson ImmunoResearch Laboratories, Inc, 1/30,000-fold dilution; HRP-conjugated goat anti-human IgG, A130PD, American Qualex, 1/30,000-fold dilution) at 25˚C for 20 min. Following five washes with TBS-T, the addition of Immobilon (Merck KGaA) produced luminescence, which was detected using LuminoGraph II (Atto Co., Ltd.), as previously described (7,8,11,17).

### Amplified luminescence proximity homogeneous assay-linked immunosorbent assay (AlphaLISA)

AlphaLISA was performed in 384-well microtiter plates (white opaque OptiPlate™, Revvity) containing either 2.5 µl of 1:100-diluted serum with 2.5 µl of GST or GST-KIAA0513 proteins (10 µg/ml) in AlphaLISA buffer (25 mM N-2-hydroxyethylpiperazine-N-2-ethane sulfonic acid, pH 7.4, 0.1% casein, 0.5% Triton X-100, 1 mg/ml dextran-500 and 0.05% Proclin-300) (Revvity). The reaction mixture was incubated at room temperature for 6-8 h, followed by the addition of anti-human IgG-conjugated acceptor beads (2.5 µl at 40 µg/ml) and glutathione-conjugated donor beads (2.5 µl at 40 µg/ml), and the mixture was incubated at room temperature in the dark for 1-14 days. The chemical emissions were measured using an EnSpire Alpha microplate reader (Revvity), as previously described (7-11). The specific reactions were calculated by subtracting the emission counts of the GST control from the counts of GST-fused KIAA0513 protein.

### Immunohistochemical staining

The formalin-fixed paraffin-embedded EC tissues were sectioned into 4-µm-thick slices, which were deparaffinized, blocked with Hyper Peroxide Block and Protein Block (Rabbit Specific HRP/DAB Detection Kit, Abcam), reacted with primary anti-KIAA0513 antibody (rabbit polyclonal antibodies, HPA012866, Atlas Antibodies) at 0.75 µg/ml for 18 h at 4˚C, incubated with biotinylated anti-rabbit IgG (biotin-conjugated goat anti-rabbit IgG, sc-2040, Santa Cruz Biotechinology, Inc.) at 2 µg/ml for 30 min at 25˚C, and reacted with streptavidin conjugated to horseradish peroxidase reagent (ab7403, Abcam) for 30 min at 25˚C. Finally, the reaction was visualized with a chromogen (diaminobenzidine) in DAB substrate (ab64238, Abcam). The sections were then counterstained with hematoxylin (Mfcd00078111, Merck KGaA) for 30 sec at 25˚C, dehydrated, mounted as previously described (11,17). Photomicrographs were obtained using a light microscope (BA210E, Shimazu) at x100 magnification.

### Nested case-control study

A nested case-cohort study was conducted using the aforementioned AlphaLISA detection antibody levels. The present study was nested within the Japan Public Health Center-based Prospective Study (50-52), which involved ~30,000 Japanese individuals aged 40-69 years at a baseline period from 1990-1994 whose plasma samples were stored. The plasma samples employed were from 202 cases of incidental AIS in the cohort that occurred between baseline and 2008 and from 202 controls whose age (within 2 years), sex, date of blood sampling (within 3 months), time since last meal (within 4 h), and study location (Public Health Center area) were matched with those of the cases. A conditional logistic regression model was used to estimate the odds ratios (ORs) and 95% confidence intervals (95% CIs). The study participants were informed of the objectives and methods of the study, and those who answered the questionnaire and donated blood were regarded as having given informed consent to participate.

### Statistical analysis

The Mann-Whitney U test was employed to determine the significant differences between two groups and the Kruskal-Wallis test (with the Bonferroni correction applied) was used to evaluate the differences among ≥3 groups. Correlations were analyzed using Spearman's correlation analysis and logistic regression analysis. All the statistical analyses were performed using GraphPad Prism 5 (GraphPad Software, Inc.). The predictive values of the putative disease markers were assessed via a receiver operating characteristic (ROC) curve analysis and determined the sensitivity and specificity. Patient survival was evaluated using the Kaplan-Meier method and compared using the log-rank test. X-tile 3.6.1 software (Yale University, New Haven, CT, USA) (53) was used to determine the optimal cut-off values for discrimination of the survival rates between antibody positive and negative groups. All tests were two-tailed, and P-values <0.05 were considered to indicate statistically significant differences.

## Results

### Recognition of KIAA0513 by serum components from patients with atherosclerosis

The present study employed a ProtoArray loaded with 9,480 protein species to identify the antigens recognized by antibodies in the sera of patients with atherosclerosis. It was found that KIAA0513 isoform c (Accession no. BC030280.1) reacted with 6 of the 10 serum samples from the patients with atherosclerosis, and with only 1 of the 10 samples from the HDs (Table SI). Subsequently, GST-fused full-length KIAA0513 protein was expressed in *E. coli* and purified by affinity-chromatography.

### Presence of autoantibodies against KIAA0513 in the sera of patients with AIS, TIA, DM, EC, or CC

**Figure 1:**
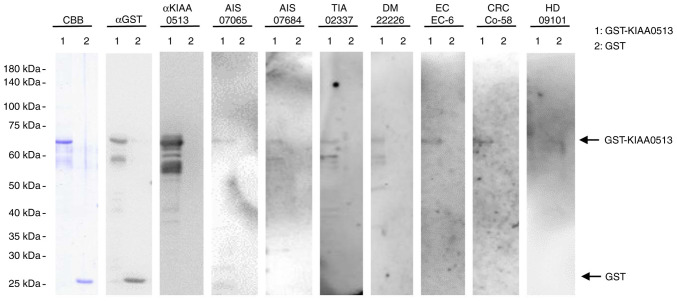
Presence of antibodies against KIAA0513 in sera from patient with AIS, TIA, DM, EC, or CRC. Purified proteins of GST-KIA0513 (lane 1) and GST (lane 2) were electrophoresed through sodium dodecyl sulfate-polyacrylamide gel, followed by staining with CBB or western blotting using anti-GST (αGST), anti-KIAA0513 (αKIAA0513), serum IgG from patients with AIS (AIS-07065, AIS-07684), TIA (TIA-02337), DM (DM-22226), EC (EC-6), or CRC (Co-58) and from a healthy donor (HD) (HD-09101). Sample number are subjects' anonymization numbers. AIS, acute ischemic stroke; TIA, transient ischemic attack; DM, diabetes mellitus; EC, esophageal cancer; CRC, colorectal cancer; CBB, Coomassie Brilliant Blue; GST, glutathione S-transferase.

The present study examined the presence of autoantibodies against KIAA0513 in sera using western blot analysis (Fig. 1). GST-KIAA0513 protein reacted with commercial anti-GST and anti-KIAA0513 antibodies, whereas the control, GST, reacted with anti-GST, but not with anti-KIAA0513 antibodies. GST-KIAA0513 protein was also recognized by serum IgG antibodies in the patients with AIS (anonymization nos. #07065 and #070684), TIA (#02337), DM (#22226), EC (#EC-6) and CRC (#Co-58), but not in the HDs (#09101). GST alone exhibited no apparent reaction with any serum from the patients or HDs.

### Elevation of s-KIAA0513-Ab levels in the patients with AIS and TIA

**Figure 2:**
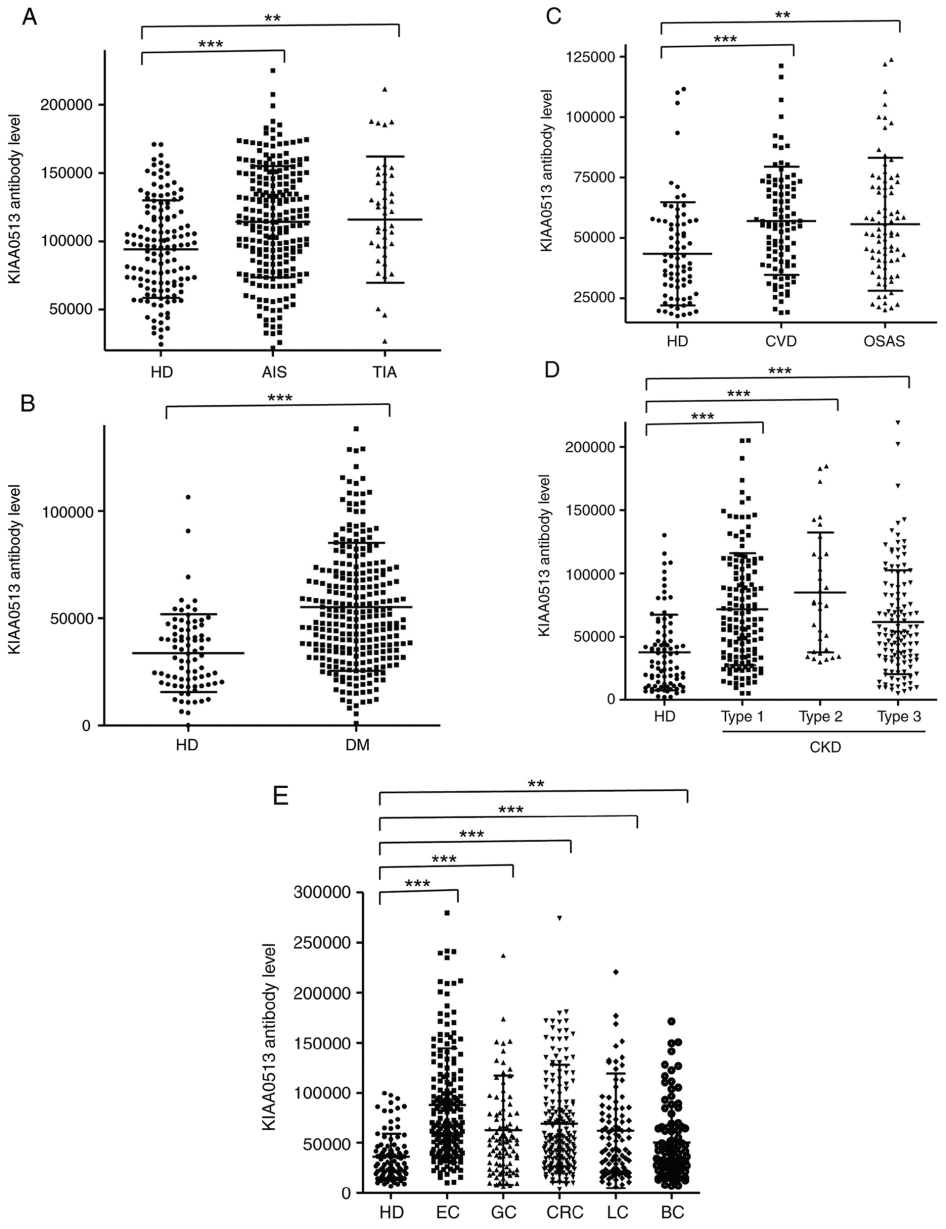
Comparison of serum anti-KIAA0513 antibody (s-KIAA0513-Ab) levels between HDs and patients. The s-KIAA0513-Ab levels of HDs and patients with (A) AIS or TIA, (B) DM, (C) CVD or OSAS, (D) CKD, and (E) EC, GC, CRC, LC, or BC were examined by AlphaLISA using GST-KIAA0513_2-302_ protein as the antigen, followed by subtraction of the levels against control GST. Scatter dot plots of the s-KIAA0513-Ab levels are shown. The bars represent the average and average ± SD. P-values were calculated using the Mann-Whitney U test to analyze the differences between two groups, and the Kruskal-Wallis test (with the Bonferroni correction applied) to evaluate the differences among ≥3 groups. ^**^P<0.01 and ^***^P<0.001 vs. HD specimens. Type-1, type-2, and type-3 CKDs represent diabetic kidney disease, nephrosclerosis, and glomerulonephritis, respectively. The total (male/female) numbers, average ages ± SDs, average antibody levels ± SDs, cut-off values, positive numbers, positive rates (%), and P-values vs. HDs are summarized and shown in Table SII, Table SIII, Table SIV, Table SV and Table VI. HD, healthy donors; AIS, acute ischemic stroke; TIA, transient ischemic attack; DM, diabetes mellitus; EC, esophageal cancer; CVD, cardiovascular disease; OSAS, obstructive sleep apnea syndrome; CKD, chronic kidney disease; GC, gastric cancer; LC, lung cancer; BC, breast cancer; SD, standard deviation.

**Figure 3:**
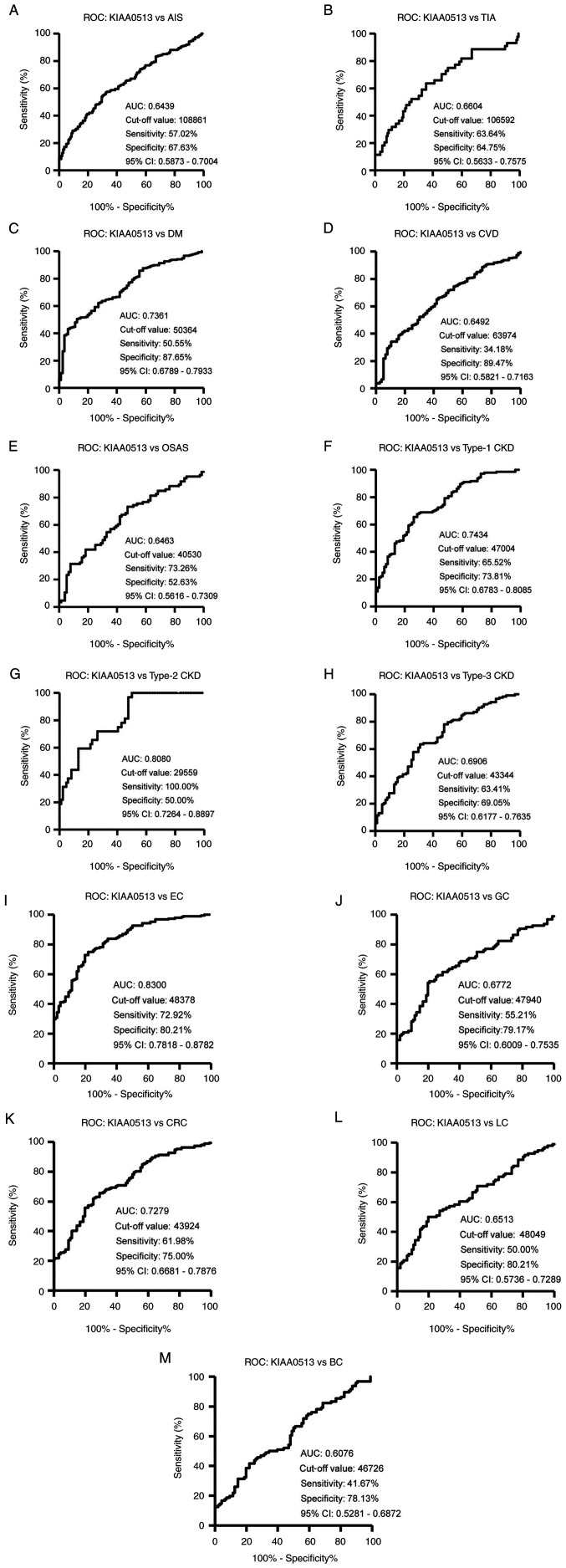
ROC curve analysis. The abilities of s-KIAA0513-Abs to detect (A) AIS, (B) TIA, (C) DM, (D) CVD, (E) OSAS, (F) type-1 CKD, (G) type-2 CKD, (H) type-3 CKD, (I) EC, (J) GC, (K) CRC, (L) LC, and (M) BC were evaluated using ROC analysis. Numbers in the figures indicate the AUC, cut-off values for antibody levels, sensitivity, specificity, and 95% CIs. ROC, receiver operating characteristic; AIS, acute ischemic stroke; TIA, transient ischemic attack; DM, diabetes mellitus; EC, esophageal cancer; CVD, cardiovascular disease; OSAS, obstructive sleep apnea syndrome; CKD, chronic kidney disease; GC, gastric cancer; LC, lung cancer; BC, breast cancer; AUC, area under the curve; CI, confidence interval.

The s-KIAA0513-Ab levels were then examined in the patients with AIS or TIA. Sera from HDs, and patients with AIS and TIA were obtained from the Chiba Prefectural Sawara Hospital. The results of AlphaLISA revealed that the s-KIAA0513-Ab levels were significantly higher in the patients with AIS or TIA than in the HDs (Fig. 2A). Using the cut-off values of the average plus two standard deviations (SDs) of the HD values, the s-KIAA0513-Ab positivity rates for the HDs, patients with AIS, and those with TIA were 0.0, 7.6 and 15.6%, respectively (Table SII). ROC analysis revealed that the areas under the ROC curves (AUCs) of s-KIAA0513-Abs were 0.6439 (95% CI, 0.587-0.700) for AIS (Fig. 3A) and 0.6604 (95% CI, 0.563-0.758) for TIA (Fig. 3B). Thus, TIA (which can be a prodromal stage of AIS) and AIS were equally associated with the s-KIAA0513-Ab marker.

### Elevation of serum antibody levels against KIAA0513 in patients with DM

The present study then examined the s-KIAA0513-Ab levels in patients with DM. Serum samples from HDs and patients with DM were obtained from Chiba University and Chiba University Hospital. The s-KIAA0513-Ab levels were significantly higher in the samples from the patients with DM than in those from HDs (Fig. 2B). At a cut-off value equivalent to the average plus two SDs of the HD specimen values, the positive rates of s-KIAA0513-Abs in the HDs and patients with DM were 2.5 and 26.5%, respectively (Table SIII). ROC analysis was performed to evaluate the ability of these antibody markers to indicate the presence of DM. The AUC for s-KIAA0513-Abs was 0.736, yielding a sensitivity and specificity of 50.55 and 87.65%, respectively (Fig. 3C).

### Association between s-KIAA0513-Ab levels and CVD and OSAS

Subsequently, the antibody levels in serum samples from patients with CVD obtained from Chiba University Hospital were examined. Given that OSAS is related to atherosclerosis and is associated with a high risk of AIS and CVD (36-39), the present study also examined the sera of patients with OSAS obtained from Chiba University Hospital. Compared with those of the HDs, the s-KIAA0513-Ab levels were significantly higher in the patients with CVD or OSAS (Fig. 2C), although the positive rates in the patients with CVD and those with OSAS were not markedly high (10.3 and 11.6%, respectively) (Table SIV). ROC analysis revealed that the AUCs for CVD and OSAS were 0.649 (95% CI, 0.582-0.716) (Fig. 3D) and 0.646 (95% CI, 0.562-0.731) (Fig. 3E), respectively. Compared with the low P-value (<0.001) of s-KIAA0513-Ab for CVD, the P-value for OSAS was <0.01 (Table SIV), suggesting a weaker association of the s-KIAA0513-Ab marker with OSAS than with CVD.

### Elevation of s-KIAA0513-Ab levels in patients with CKD

The present study then examined the antibody levels in the sera of patients with CKD, which is also closely related to atherosclerosis. CKD was divided into three groups as follows: Type 1, diabetic kidney disease; type 2, nephrosclerosis; and type 3, glomerulonephritis. Samples from patients with CKD were obtained from the Kumamoto cohort, and samples from HDs were obtained from Chiba University. Patients from all three CKD groups had significantly higher s-KIAA0513-Ab levels than the HDs (Fig. 2D). The s-KIAA0513-Ab positivity rates in the HDs and patients with types 1, 2 and 3 CKD were 6.1, 29.0, 37.5 and 20.3%, respectively (Table SV), indicating that the highest positive rate was observed in the patients with type 2 CKD. ROC analysis revealed s-KIAA0513-Ab AUCs as high as 0.7434 (95% CI, 0.678-0.809) for type 1 CKD (Fig. 3F), 0.808 (95% CI, 0.726-0.890) for type 2 CKD (Fig. 3G) and 0.691 (95% CI, 0.618-0.764) for type 3 CKD (Fig. 3H).

### s-KIAA0513-Ab levels in solid cancer

Given that atherosclerotic diseases are frequently related to cancer with certain common biomarkers being reported (20), the present study examined the serum samples from patients with EC, GC, CRC, LC and BC obtained from Toho University Hospital. The s-KIAA0513-Ab levels were significantly higher in the samples from all patients with cancer than in those from the HDs (Fig. 2E and Table SVI). The highest average value and positive rate of s-KIAA0513-Ab levels were observed for EC. Similarly, the AUC values were highest for EC (0.830), but lowest for BC among the cancers examined (Fig. 3I-M).

**Figure 4:**
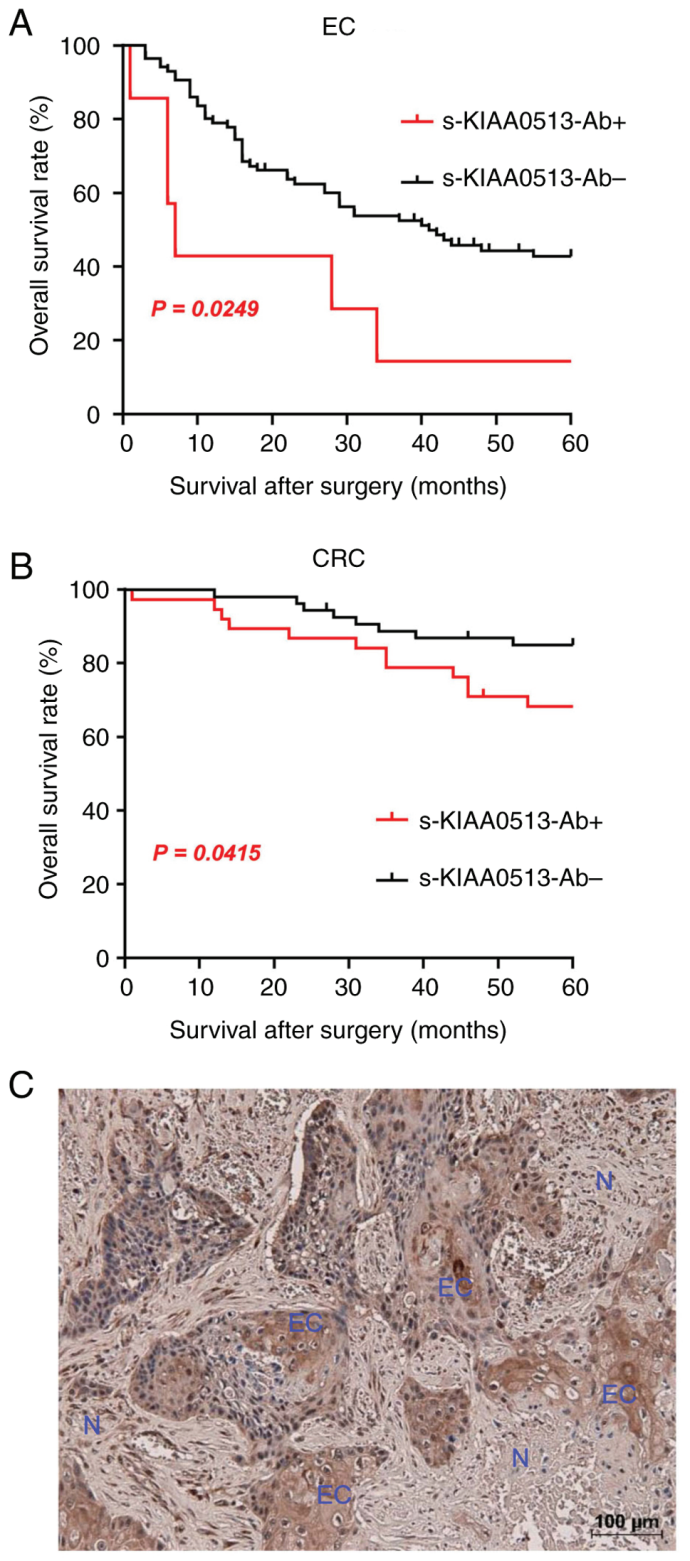
Survival analysis and immunohistochemical staining of cancer specimens. Comparison of overall survivals of the patients with (A) EC and (B) CRC according to s-KIAA0513-Ab-positive (s-KIAA0513-Ab^+^) and negative (s-KIAA0513-Ab^-^) groups. Cut-off values of EC and CRC were determined as 180,487 and 56,879, respectively, using X-tile analysis. Statistical analyses were performed using the log-rank test. (C) Surgically resected EC tissue was stained using anti-KIAA0513 antibody (rabbit polyclonal antibodies, Atlas Antibodies). EC and N represent cancerous and normal cells, respectively. EC, esophageal cancer; CRC, colorectal cancer.

The present study then examined whether the s-KIAA0513-Ab levels are related to the post-operative survival of patients with EC or GC. The s-KIAA0513-Ab levels were divided into the positive and negative groups using the cut-off values obtained using X-tile software (53). The s-KIAA-Ab-positive group presented a more unfavorable prognosis than the negative group in all of EC and CRC (Fig. 4A and B). The X-tile-determined cut-off values are best ones to distinguish the favorable and poor survivals. The cut-off value of EC samples (180,487) was much higher than the average (87,535) (Table SVI), whereas that of CRC (56,879) was lower than the average of CRC (69,308) (Table SVI). Thus, the considerably high levels of s-KIAA-Abs in patients with EC and the moderately high levels in patients with CRC were associated with the prognosis.

The expression levels of KIAA0513 antigenic protein in EC tissues were examined using immunohistochemical staining. A representative example of the staining is illustrated in Fig. 4C. EC tissues were heavily stained by anti-KIAA0513 antibody, whereas surrounding healthy esophageal tissues were not. KIAA0513 protein was localized in the cytoplasm, which is consistent with the findings in a previous study (54). Thus, the high KIAA0513 expression levels may account for some, if not all, of the development of serum KIAA0513-Abs.

### Association analysis

An analysis of the association analysis between the s-KIAA0513-Ab levels and participant data was performed using 665 specimens from Chiba Prefectural Sawara Hospital, including 139 specimens from HDs, 225 from patients with AIS, 44 from patients with TIA, 17 from patients with Asympt-CI, 122 from patients with DSWMH, 59 from patients with cCI and 41 from disease controls. The remaining 18 subjects were excluded because they did not have disease information. In this analysis, the Mann-Whitney U test we employed to compare the s-KIAA0513-Ab levels between the male and female participants, with or without DM, hypertension, CVD, dyslipidemia and obesity [body mass index (BMI) ≥25] and with or without smoking and alcohol intake habits. A significant difference in the s-KIAA0513-Ab levels was observed only between the patients with hypertension and those without hypertension (Table I).

### Correlation analysis

Spearman's correlation analysis was performed a to determine the correlation between the s-KIAA0513-Ab levels and the continuous variables of participant parameters, including general information such as age, height, weight and BMI; the degree of artery stenosis, such as the maximum intima-media thickness (max-IMT); lifestyle factors such as smoking duration (years) and alcohol intake frequency (times/week); and blood test data. The average values of these parameters are listed in Table SVII. There was a significant correlation between the s-KIAA013-Ab levels and age, max-IMT, alkaline phosphatase, potassium, C-reactive protein (CRP), blood sugar and smoking duration (Table II), and an inverse correlation with height and weight. The correlation with max-IMT suggests that the s-KIAA0513-Ab levels are associated with stenosis and atherosclerosis, which was further confirmed by using other cohorts. Spearman's correlation analysis of the CKD cohort (300 participants) revealed a significant correlation with plaque score, max-IMT (55,56) and cardio-ankle vascular index (CAVI) (right) (57) (Table SVIII), which are indices of atherosclerosis. CRP was also associated with s-KIAA0513-Abs in the CKD cohort, suggesting the involvement of inflammation. By contrast, age, height, weight, BMI and potassium levels exhibited no significant correlation with s-KIAA0513-Abs in the CKD cohort. AIS is closely related to age, which may be indirectly associated with s-KIAA0513-Abs.

### Japan Public Health Center (JPHC) cohort analysis

A case-control study nested within the Japan Public Health Center-based Prospective Study was then conducted, which involved ~30,000 plasma samples (50-52). The level of antibodies against the KIAA0513 protein was positively associated with the risk of AIS. The ORs (95% CIs) were 2.11 (1.17-3.81) and 2.23 (1.18-4.21) for those in the third and highest quartiles of antibody levels, respectively, compared with those in the lowest quartile (Table III). These results indicate that the antibody markers against the KIAA0513 protein are useful for predicting the onset of AIS.

## Discussion

In the present study, the initial ProtoArray screening identified KIAA0513 as an antigen, as recognized by serum IgG in patients with atherosclerosis, and subsequently recombinant GST-tagged KIAA0513 protein of 301 amino acids was purified. Western blot analysis confirmed the presence of autoantibodies against KIAA0513 (Fig. 1). Using the KIAA0513 protein as an antigen, the serum antibody levels were examined using AlphaLISA. The results revealed significantly higher s-KIAA0513-Ab levels in the patients with AIS, TIA, DM, CVD, OSAS, CKD, EC, GC, CC, LC and MC than in the HDs (Fig. 2A-E and Table SII, Table SIII, Table SIV, Table SV and Table VI). Among these diseases, the highest AUC values were observed for EC, type 2 CKD and DM (Fig. 3A-M). The close association between s-KIAA0513-Ab levels and hypertension (Table I) could account for the association with OSAS, which is frequently accompanied by hypertension (58). Spearman's correlation analysis revealed a significant correlation between s-KIAA0513-Ab and max-IMT, plaque score and CAVI, all of which are indices of atherosclerosis-related lesions (Tables II and SVIII) (55-57). By contrast, the s-KIAA0513-Ab levels were weakly correlated with blood sugar (P=0.023), but were completely unrelated to HbA1c, a typical DM marker (Table II). Thus, although the patients with DM exhibited high s-KIAA0513-Ab levels compared with the HDs, this antibody marker may not primarily reflect DM lesions, but rather atherosclerotic lesions caused by DM. Given that angiogenesis is essential for the development of cancer, vascular malformation may be accompanied by the typical alterations in atherosclerosis. In fact, DM and arteriosclerotic diseases are cancer risk factors (59-61).

There are three known splicing variants of KIAA0513: Isoform a (411 amino acids, NP_055547.1), isoform b (301 amino acids, NP_001273495.1) and isoform c (301 amino acids, NP_001284695.1). The full-length 301 amino acids of KIAA0513 isoforms c and b are exactly the same as the first 301 amino acids of KIAA0513 isoform a. The present study also purified GST-fused KIAA0513 isoform a and examined the serum antibodies using sera from HDs and patients with AIS and CVD. Both isoforms a and c of KIAA0513 exhibited higher antibody levels in the sera from patients with AIS or CVD than in the sera from HDs (Fig. S1A and B). The reactivity of KIAA0513 isoform c against serum antibodies was closely associated with that of KIAA0513 isoform a, although the former was higher than the latter (Fig. S1C), implying that the major epitope sites of serum autoantibodies are located in the 301 amino acids of isoform c.

KIAA0513 mRNA expression has been observed predominantly in the neurons and glial cells of the brain, with low-level expression in most human tissues, whereas the KIAA0513 protein was exclusively found in the brain (54). Among brain regions, the highest expression was in the cerebellum, cortex, hippocampus, pons, putamen and amygdala. Using a yeast 2-hybrid analysis of a fetal brain cDNA library, Lauriat *et al* (54) found that the N-terminal portion of KIAA0513 interacted with KIBRA, HAX1 and INTS4. A coimmunoprecipitation analysis revealed a physical association between KIAA0513 and KIBRA. Given that KIBRA, HAX1 and INTS4 are involved in synaptic and apoptotic signaling, KIAA0513 can also participate in these signaling pathways.

In addition to the KIAA0513-Abs employed in the present study, autoantibodies against ATPase, Ca^++^ transporting, plasma membrane 4, bone morphogenetic protein 1, deoxyhypusine synthase, low-density lipoprotein receptor-related protein-associated protein 1 and additional sex combs-like 2, which are markers of atherosclerosis, were also elevated in the sera of patients with EC (13,14,17,18), indicating that arterial abnormalities can also affect the carcinogenic process. In fact, angiogenesis is essential for the development of solid tumors (62), and diabetes and obesity, which induce arteriosclerosis, are risk factors for CRC and EC (63-65). Given that all tissues and organs require oxygen and nutrition provided by arteries, the alteration of arterial structure and/or function can affect numerous tissues and organs. All tissues and organs present in a body can affect each other to a certain degree (66). In other words, the AIS, CVD and CKD caused by atherosclerosis, the atherosclerosis induced by DM, and the solid cancer caused by arterial lesions can be interrelated with each other via arterial abnormalities. Markers associated with such abnormalities could therefore detect all of the above disorders.

Cancer, heart disease, cerebrovascular disease and renal failure are the first, second, fourth and eighth leading causes of mortality in Japan, respectively (Ministry of Health, Labor and Welfare 2018 vital statistics; https://www.mhlw.go.jp/toukei/saikin/hw/jinkou/kakutei18/dl/10_h6.pdf). The majority of the other causes of death are unavoidable, such as senility and accidents. In other words, the onset and progression of cancer, heart disease, cerebrovascular disease and renal failure (as well as their risk factor DM) can be suppressed by proper health management, such as early diagnosis and intervention. Notably, cancer, heart disease, cerebrovascular disease, renal failure and DM can be detected by the s-KIAA0513-Ab marker, making it applicable for diagnostic purposes and providing appropriate treatment, lifestyle guidance, etc., leading to improved quality of life.

As of 2020, numerous reports have shown that the presence of underlying diseases, such as DM, heart disease, cerebrovascular disease, cancer, OSAS and kidney disease aggravate the coronavirus disease 2019 (COVID-19) (67-70). The s-KIAA0513-Ab marker is therefore a highly useful tool for detecting patients with COVID-19 who are at a higher risk of mortality. Antibody markers are generally more sensitive than antigen markers. Given that the KIAA0513 protein has particularly high antigenicity, this KIAA0513-Ab marker is extremely sensitive. Given the major life-threatening diseases can be detected by this marker, the KIAA0513-Ab marker could be referred to as a ‘supermarker’.

The present study has certain limitations, which should be mentioned. First, although the increase in s-KIAA0513-Ab levels could be attributable to the high KIAA0513 expression levels (Fig. 4C) as suggested above, the association between the expression of the antigen and the antibody has not been completely verified. The antigen levels can be examined by immunohistochemistry, western blot analysis and mass spectrometry. However, accurately quantifying protein amounts across many specimens using these methods is still challenging. The introduction of the AlphaLISA method for the quantification of antigenic proteins may be another approach. Second, the significant differences of the prognosis between the s-KIAA-Ab-positive and -negative groups were observed in EC and CRC (Fig. 4A and B) but not GC, LC, or BC. Further accumulation of the latter specimens may clarify the association between s-KIAA0513-Ab levels and their prognosis.

In conclusion, the serum anti-KIAA0513 antibody marker appears to be useful for diagnosing the progress of atherosclerosis, which can lead to the onset of life-threatening AIS, CVD and cancer.

## Supplementary Material

Comparison of reactivity between isoform c (amino acids 2-302) and isoform a (amino acids 1-411) of KIAA0513 as antigens for evaluation of serum antibody levels. The s-KIAA0513-Ab levels of HDs and patients with AIS or CVD were examined by AlphaLISA using GST-KIAA05132-302 (A) and GST-KIAA05131-411 (B) proteins as the antigens. A scatter dot plot of the antibody levels is shown. Results are presented as described in the legend of Fig. 2. ^***^P<0.001 vs. HD specimens. The bars represent the average ± SD. (C) Correlation plot of antibody levels against KIAA0513 isoform a vs. isoform c.

ProtoArray^®^ screening for autoantibodies in atherosclerosis.

Comparison of the serum antibody levels of HDs vs. those of patients with AIS or TIA.

Comparison of the serum antibody levels of HDs vs. those of patients with DM.

Comparison of the serum antibody levels of HDs vs. those of patients with CVD or OSAS.

Comparison of s-KIAA0513-Ab levels of HDs vs. those of patients with CKD.

Comparison of s-KIAA0513-Ab levels between HDs and patients with cancer.

Information of subjects in the Sawara Hospital cohort used for correlation analysis.

Correlation analysis between serum KIAA0513-Ab levels and the data of CKD cohort.

## Figures and Tables

**Table I:** Association analysis of antibody levels against KIAA0513 protein with data of subjects in the Sawara Hospital cohort.

Sex	Average or SD	Male	Female
No. of samples		396	268
KIAA0513-Ab level	Average	100,136	104,138
	SD	39,889	40,969
P-value (vs. male)			0.1466
Other disease		DM^-^	DM^+^
No. of samples		525	135
KIAA0513-Ab level	Average	101,818	101,727
	SD	40,022	41,930
P-value (vs. DM^-^)			0.5031
Other disease		Hypertension^-^	Hypertension^+^
No. of samples		239	421
KIAA0513-Ab level	Average	96,476	104,821
	SD	38,988	40,905
P-value (vs. hypertension^-^)			**0.0091**
Other disease		CVD^-^	CVD^+^
No. of samples		623	37
KIAA0513-Ab level	Average	101,376	108,939
	SD	39,757	49,697
P-value (vs. CVD^-^)			0.1177
Other disease		Lipidemia^-^	Lipidemia^+^
No. of samples		475	185
KIAA0513-Ab level	Average	103,028	98,647
	SD	40,591	39,808
P-value (vs. lipidemia^-^)			0.2716
Lifestyle		Non-smoker	Smoker
No. of samples		344	319
KIAA0513-Ab level	Average	98,991	104,832
	SD	38,563	42,055
P-value (vs. non-smoker)			0.0673
Lifestyle		Alcohol^-^	Alcohol^+^
No. of samples		238	419
KIAA0513-Ab level	Average	102,663	101,364
	SD	39,883	40,759
P-value (vs. alcohol^-^)			0.4595
Obesity		BMI <25	BMI >25
No. of samples		498	158
KIAA0513-Ab level	Average	102,071	101,390
	SD	40,475	40,488
P-value (vs. alcohol^-^)			0.7490

The subjects were divided as follows: Sex (male and female); presence (+) or absence (-) of complication of DM, hypertension, CVD, or dyslipidemia, and lifestyle factors (smoking and alcohol intake habits, and obesity). Antibody levels (Alpha counts) were compared using the Mann-Whitney U test. Sample numbers, averages and SDs of counts, as well as P-values are shown. Significant associations (P<0.05) are indicated in bold font. BMI, body mass index; DM, diabetes mellitus; CVD, cardiovascular disease.

**Table II:** Correlation analysis of serum antibody levels against KIAA0513 with data on subjects in the Sawara Hospital cohort.

		KIAA0513-Ab	
Parameter	No. of XY pairs	Rs value	P-value
Age^a^	663	0.1959	**<0.0001^b^**
Height	657	-0.1317	**0.0007**
Weight	661	-0.1093	**0.0049**
BMI	656	-0.0414	0.2899
max-IMT	457	0.1763	**0.0002**
A/G	628	-0.0187	0.6402
AST (GOT)	655	0.0395	0.3134
ALT (GPT)	655	-0.0035	0.9278
ALP	600	0.0889	**0.0295**
LDH	631	0.0431	0.2798
tBil	637	0.0317	0.4250
CHE	506	-0.0387	0.3851
γ-GTP	609	-0.0099	0.8072
TP	633	-0.0184	0.6435
ALB	638	-0.0207	0.6014
BUN	654	-0.0454	0.2469
CRE	649	-0.0310	0.4303
eGFR	552	0.0432	0.3113
UA	492	-0.0138	0.7596
AMY	415	-0.0901	0.0667
T-CHO	563	-0.0446	0.2913
HDL-C	435	-0.0280	0.5600
TG	460	-0.0198	0.6719
Na	641	-0.0347	0.3808
K	641	-0.0899	**0.0228**
Cl	641	-0.0392	0.3215
Ca	380	-0.0607	0.2376
IP	302	-0.0085	0.8824
Fe	311	-0.0083	0.8838
CRP	477	0.0917	**0.0453**
LDL-C	344	-0.0559	0.3011
WBC	650	0.0712	0.0697
RBC	650	-0.0220	0.5748
HGB	650	0.0117	0.7651
HCT	650	-0.0041	0.9174
MCV	650	0.0525	0.1814
MCH	650	0.0611	0.1199
MCHC	650	0.0401	0.3080
RDW	650	0.0437	0.2661
PLT	650	-0.0269	0.4942
MPV	650	-0.0354	0.3683
PCT	650	-0.0271	0.4900
PDW	650	-0.0342	0.3838
Blood sugar	596	0.0932	**0.0229**
HbA1c	505	-0.0212	0.6351
Smoking duration, years	656	0.1297	**0.0009**
Alcohol frequency, times/week	662	-0.0328	0.4000

Sample numbers, correlation coefficients (Rs values) and P-values obtained by Spearman's correlation analysis are shown.

^a^Subject data used are listed in the first column.

^b^Significant correlations (P<0.05) are indicated in bold font. BMI, body mass index; max-IMT, maximum intima-media thickness; A/G, albumin/globulin ratio; AST, aspartate aminotransferase; ALT, alkaline aminotransferase; ALP, alkaline phosphatase; LDH, lactate dehydrogenase; tBil, total bilirubin; CHE, cholinesterase; γ-GTP, γ-glutamyl transpeptidase; TP, total protein; ALB, albumin; BUN, blood urea nitrogen; CRE, creatinine; eGFR, estimated glomerular filtration rate; UA, uric acid; AMY, amylase; T-CHO, total cholesterol; HDL-C, high-density lipoprotein cholesterol; TG, triglyceride; Na, sodium; K, potassium; Cl, chlorine; Ca, calcium; IP, inorganic phosphate; Fe, iron; CRP, C-reactive protein; LDL-C, low-density lipoprotein cholesterol; WBC, white blood cell number; RBC, red blood cell number; HGB, hemoglobin; HCT, hematocrit; MCV, mean corpuscular volume; MCH, mean corpuscular hemoglobin; MCHC, MCH concentration; RDW, red cell distribution width; PLT, platelet number; MPV, mean platelet volume; PCT, procalcitonin; PDW, platelet distribution width; HbA1c, glycated hemoglobin.

**Table III:** Odds ratios of incident KIAA0513-Abs vs. AIS.

Quartile group	OR and 95% CI	KIAA0513-Ab vs. AIS
2nd	Matched OR	1.69
	95% CI	0.91-3.15
3rd	Matched OR	2.11
	95% CI	1.17-3.81
Max	Matched OR	2.23
	95% CI	1.18-4.21

Results of a case-control study nested within the Japan Public Health Center-based Prospective Study are shown. The ORs and 95% CIs of the 2nd, 3rd, and the highest (max) quartiles vs. the lowest quartile are shown for AIS with respect to the antibody levels of s-KIAA0513-Abs. OR, odds ratio; CI, confidence interval.

## Data Availability

All data of the ProtoArray v4.0 human protein microarray system are available in the public Figshare database (https://figshare.com/articles/dataset/Results_of_protein_array_for_atherosclerosis/25906330). The other datasets used and/or analyzed during the current study are available from the corresponding author on reasonable request.
