# Supplementary material for: Serum anti‑KIAA0513 antibody as a common biomarker for mortal atherosclerotic and cancerous diseases
Source: Med Int (Lond). 2024 Jun 19;4(5):45. doi: 10.3892/mi.2024.169 (PMC11228693; doi:10.3892/mi.2024.169)

Figure S1. Comparison of reactivity between isoform c (amino acids 2-302) and isoform a (amino acids 1-411) of KIAA0513 as antigens for evaluation of serum antibody levels. The s-KIAA0513-Ab levels of HDs and patients with AIS or CVD were examined by AlphaLISA using GST-KIAA0513$_{2-302}$ (A) and GST-KIAA05131-411 (B) proteins as the antigens. A scatter dot plot of the antibody levels is shown. Results are presented as described in the legend of Fig. 2. $^{***}P<0.001$ vs. HD specimens. The bars represent the average ± SD. (C) Correlation plot of antibody levels against KIAA0513 isoform a vs. isoform c.

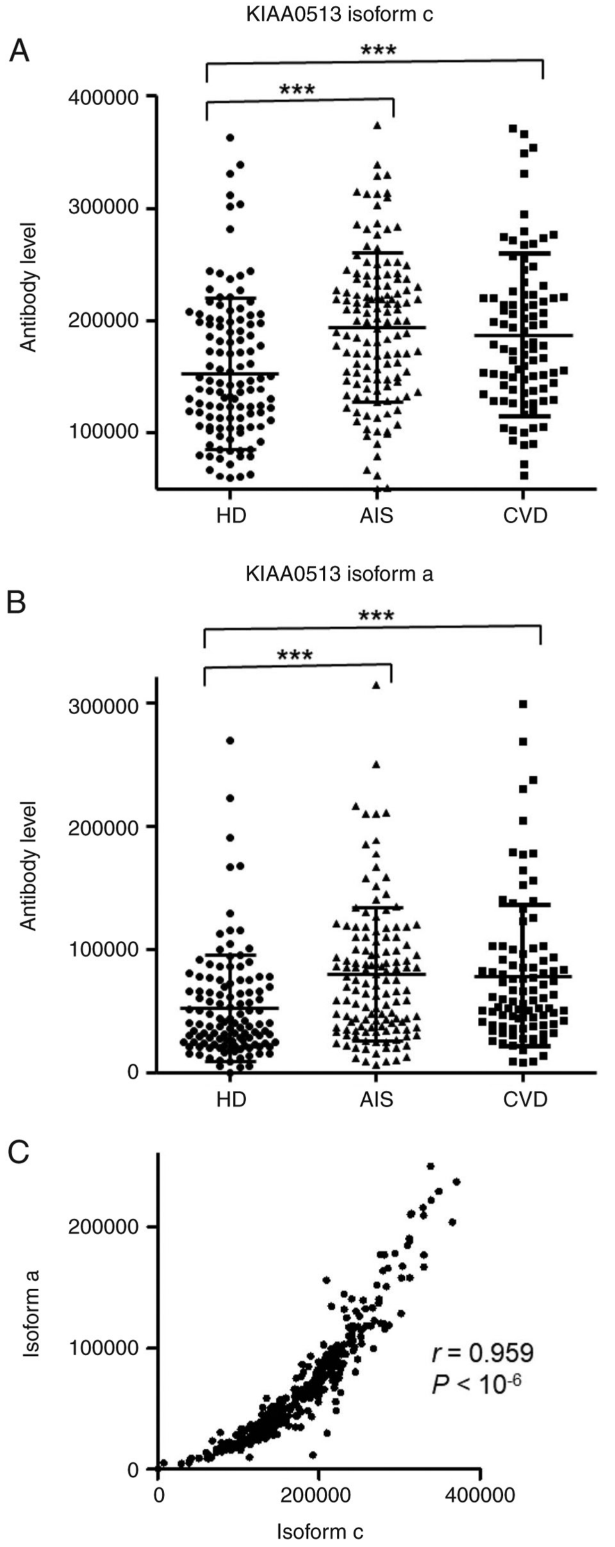

Supplement: Comparison of reactivity between isoform c (amino acids 2-302) and isoform a (amino acids 1-411) of KIAA0513 as antigens for evaluation of serum antibody levels. The s-KIAA0513-Ab levels of HDs and patients with AIS or CVD were examined by AlphaLISA using GST-KIAA05132-302 (A) and GST-KIAA05131-411  [file Supplementary_Data1.pdf]
