# Supplementary material for: Serum anti‑KIAA0513 antibody as a common biomarker for mortal atherosclerotic and cancerous diseases
Source: Med Int (Lond). 2024 Jun 19;4(5):45. doi: 10.3892/mi.2024.169 (PMC11228693; doi:10.3892/mi.2024.169)
Supplement: Comparison of the serum antibody levels of HDs vs. those of patients with AIS or TIA. [file Supplementary_Data3.pdf]

Table SII. Comparison of the serum antibody levels of HDs vs. those of patients with AIS or TIA.

A, Sample information

	Subject group		
Information	HD	AIS	TIA
Total no. of samples	139	228	44
Male/female	87/52	129/99	24/20
Age (years), average ± SD	51.6±12.8	77.0±11.1	68.5±12.1

B, Serum antibody levels (Alpha photon counts)[a]

Subject group	Type of value	KIAA0513-Ab
HD	Average	92,849
	SD	36,115
	Cut-off value	165,080
	Positive no.	0
	Positive (%)	0.0%
AIS	Average	110,913
	SD	40,351
	Positive no.	11
	Positive (%)	7.6%
	P-value (AIS vs. HD)	**<0.001**
TIA	Average	122,303
	SD	42,845
	Positive no.	5
	Positive (%)	**15.6%**
	P-value (TIA vs. HD)	<0.01

[a]The serum antibody levels (Alpha photon counts) were examined by AlphaLISA using purified KIAA0513-GST protein as an antigen. Cut-off values were determined as the average HD values plus two SD, and positive samples higher than the cut-off value were scored. P-values were calculated using the Kruskal-Wallis test. P values <0.05 and positive rates >10% are indicated in bold font. A scatter dot plot of the same results is presented in Fig. 2A. HD, healthy donor; AIS, acute ischemic stroke; TIA, transient ischemic attack; SD, standard deviation.

Table SIII. Comparison of the serum antibody levels of HDs vs. those of patients with DM.

A, Sample information

	Subject group	
Information	HD	DM
Total no. of samples	81	275
Male/female	46/35	158/117
Type 1 DM/type 2 DM	-	26/216[a]
Age (years),average ± SD	45.2±11.0	63.1±12.0

B, Serum antibody levels[b]

Subject group	Type of value	KIAA0513-Ab
HD	Average	33,698
	SD	18,121
	Cut-off value	69,941
	Positive no.	2
	Positive (%)	2.5%
DM	Average	55,282
	SD	29,897
	Positive no.	73
	Positive (%)	26.5%
	P-value(vs. HD)	**<0.001**

[a]33 Patients had undetermined DM. [b]The serum antibody levels were examined by AlphaLISA using purified KIAA0513-GST protein as an antigen, as described in the legend of Table SII.P-values were calculated using the Mann-Whitney U test. P-values <0.05 and positive rates >10% are indicated in bold font. A scatter dot plot of the same results is presented in Fig. 2B. DM, diabetes mellitus; SD, standard deviation.

Table SIV. Comparison of the serum antibody levels of HDs vs. those of patients with CVD or OSAS.

A, Sample information

	Subject group		
Sample information	HD	CVD	OSAS
Total no. of samples	76	97	86
Male/female	43/33	81/16	59/27
Age (years), average ± SD	45.1±11.5	66.3±11.4	57.8±12.5

B, Serum antibody levels[a]

Subject group	Type of value	KIAA0513-Ab
HD	Average	43,398
	SD	21,374
	Cut-off value	86,146
	Positive no.	4
	Positive (%)	5.3%
CVD	Average	57,531
	SD	22,460
	Positive no.	10
	Positive (%)	**10.3%**
	P-value (CVD vs. HD)	**<0.001**
OSAS	Average	55,650
	SD	27,493
	Positive no.	10
	Positive (%)	**11.6%**
	P-value (OSAS vs. HD)	**<0.01**

[a]The serum antibody levels (Alpha photon counts) were examined by AlphaLISA using purified KIAA0513-GST protein as an antigen as described in the legend of Table SII. Cut-off values were determined as the average HD values plus two SD, and positive samples higher than the cut-off value were scored. P-values were calculated using the Kruskal-Wallis test. P-values <0.05 and positive rates >10% are indicated in bold font. A scatter dot plot of the same results is presented in Fig. 2C. HD, healthy donor; CVD, cardiovascular disease; OSAS, obstructive sleep apnea syndrome; SD, standard deviation.

Table SV. Comparison of s-KIAA0513-Ab levels of HDs vs. those of patients with CKD.

A, Sample information

	Subject group			
Sample information	HD	Type-1 CKD	Type-2 CKD	Type-3 CKD
Total no. of samples	82	145	32	123
Male/female	44/38	106/39	21/11	70/53
Age (years), average ± SD	44.1±11.2	66.0±10.4	76.0±9.8	62.0±11.7

B, Serum antibody levels[a]

Subject group	Type of value	KIAA0513-Ab
HD	Average	36,007
	SD	28,697
	Cut-off value	93,400
	Positive no.	5
	Positive rate (%)	6.1%
Typ-1 CKD	Average	71,773
	SD	44,434
	Positive no.	42
	Positive rate (%)	**29.0%**
	P-value (vs. HD)	**<0.001**
Type-2 CKD	Average	84,954
	SD	47,414
	Positive no.	12
	Positive rate (%)	**37.5%**
	P-value (vs. HD)	**<0.001**
Type-3 CKD	Average	61,443
	SD	41,110
	Positive no.	25
	Positive rate (%)	**20.3%**
	P-value (vs. HD)	**<0.001**

[a]The serum antibody levels (Alpha photon counts) were examined by AlphaLISA using purified KIAA0513-GST protein as an antigen as described in the legend of Table SII. Cut-off values were determined as the average HD values plus two SD, and positive samples higher than the cut-off value were scored. P-values were calculated using the Kruskal-Wallis test. P-values <0.05 and positive rates >10% are indicated in bold font. A scatter dot plot of the same results is presented in Fig. 2D. HD, healthy donor; CKD, chronic kidney disease; SD, standard deviation.

Table SVI. Comparison of s-KIAA0513-Ab levels between HDs and patients with cancer.

A, Sample information

	Subject group					
Sample information	HD	EC	GC	CC	LC	MC
Total no. of samples	96	192	96	192	96	96
Male/female	51/45	155/37	68/28	119/74	0/96	58/38
Age (years), average ± SD	57.9±6.0	67.4±9.8	68.7±10.6	66.7±11.7	60.9±13.3	68.1±9.6

B, Serum antibody levels[a]

Subject group	Type of value	KIAA0513-Ab
HD	Average	36,199
	SD	23,001
	Cut-off value	82,201
	Positive no.	8
	Positive (%)	8.3%
EC	Average	87,535
	SD	57,134
	Positive no.	86
	Positive (%)	**44.8%**
	P-value (EC vs. HD)	**<0.001**
GC	Average	62,714
	SD	54,540
	Positive no.	21
	Positive (%)	**21.9%**
	P-value (GC vs. HD)	**<0.001**
CRC	Average	69,308
	SD	58,449
	Positive no.	53
	Positive (%)	**27.6%**
	P-value (CRC vs. HD)	**<0.001**
LC	Average	62,148
	SD	57,101
	Positive no.	24
	Positive (%)	**25.0%**
	P-value (LC vs. HD)	**<0.001**
BC	Average	50,270
	SD	36,802
	Positive no.	18
	Positive (%)	**18.8%**
	P-value (MC vs. HD)	**0.002**

[a]The serum antibody levels (Alpha photon counts) were examined by AlphaLISA using purified KIAA0513-GST protein as an antigen as described in the legend of Table SII. Cut-off values were determined as the average HD values plus two SD, and positive samples higher than the cut-off value were scored. P-values were calculated using the Kruskal-Wallis test. P-values <0.05 and positive rates >10% are indicated in bold font. A scatter dot plot of the same results is presented in Fig. 2E. HD, healthy donor;EC, esophageal cancer;GC, gastric cancer;CRC, colorectal cancer; LC, lung cancer; BC, breast cancer.

Table SVII. Information of subjects in the Sawara Hospital cohort used for correlation analysis.

Information	Subject information	Average ± SD
General	Age, years	67.75±14.80[a]
	Sex (male/female)	395/270
	Body height	158.55±9.94 (cm)
	Body weight	58.03±12.22 (kg)
	Body mass index	22.94±3.59
Artery stenosis	Maximum intima-media thickness	2.37±1.34 (mm)
Lifestyle habits	Smoking habit duration	16.2±20.4 (year)
	Alcohol intake frequency	2.22±2.98 (times/week)
Blood test results	LDL-cholesterol	115.6±28.9 (mg/d;)
	Alkaline phosphatase	244.3±90.7 (U/;)
	Total cholesterol	196.4±35.7 (mg/d;)
	Chlorine	104.6±3.1 (mEq;)
	HDL-cholesterol	55.5±15.4 (mg/d;)
	Potassium	4.16±0.41 (mq/;)
	Creatinine	0.80±0.32 (mg/dl)
	Gamma-glutamyl transpeptidase	35.0±43.6 (U/l)
	Uric acid	5.79±9.73 (mg/dl)
	Hemoglobin A1c	5.82±2.28 (%)
	Albumin	4.26±0.48 (g/dl)
	Total Protein	7.19±0.54 (g/dl)
	Sodium	140.3±2.5 (mEq/l)
	Alanine aminotransferase	21.4±15.6 (U/l)
	Triglycerides	123.9±84.1 (mg/dl)
	Estimated glomerular filtrating ratio	73.2±21.6 (mL/min/1.73m^2)
	Aspartate aminotransferase	24.54±17.43 (U/l)
	Cholinesterase	319.9±79.8 (U/l)
	Blood urea nitrogen	15.68±9.14 (mg/dl)
	Total bilirubin	0.13±0.14 (mg/dl)
	Blood sugar	125.0±45.4 (mg/dl)
	Lactate dehydrogenase	198.9±118.8 (U/l)
	Albumin/globulin ratio	1.49±0.29

[a]Average ± SD values of the specimens in each category are shown. LDL, low-density lipoprotein; HDL, high-density lipoprotein.

Table SVIII. Correlation analysis between serum KIAA0513-Ab levels and the data of CKD cohort.

Parameter	Rs value	P-value[c]
Age[a]	0.0640	0.2695
Height	0.0690	0.2342
Weight	-0.0180	0.7567
BMI	-0.0736	0.2045
Plaque score	0.2306	**<0.0001**
Max-IMT	0.1157	**0.0468**
CAVI (right)	0.1244	**0.0371**
CAVI (left)	0.1133	0.0568
ABI (right)	-0.0164	0.7804
ABI (left)	-0.0559	0.3389
HbA1c	-0.0890	0.2821
W-PTH	0.0502	0.3864
Dialysis period[b]	-0.0176	0.7614
ARB	-0.0294	0.6116
ACE	0.0977	0.0910
PTA	0.1276	**0.0273**
Fe	-0.0456	0.4319
Ferritin	0.1655	**0.0040**
TSAT ratio	0.0399	0.4910
Kt/V	-0.0746	0.1975
RBC	-0.0801	0.1664
HGB	-0.0518	0.3713
HCT	-0.0322	0.5787
PLT	-0.1867	**0.0012**
TP	-0.0368	0.5259
ALB	-0.1337	**0.0205**
UN	-0.1303	**0.0240**
CRE	-0.0645	0.2655
UA	-0.0570	0.3256
Na	0.0830	0.1515
K	-0.0043	0.9405
Cl	0.0494	0.3936
Ca	0.0039	0.9459
IP	-0.0242	0.6768
Mg	0.0675	0.2436
AST	0.0816	0.1586
ALT	0.0111	0.8486
LDH	0.0591	0.3076
γ-GTP	0.0811	0.1610
ALP	0.0031	0.9574
tBil	0.0344	0.5530
AMY	-0.0151	0.7940
CK	-0.0239	0.6799
T-Chol	-0.0655	0.2583
HDL-C	-0.1534	**0.0078**
LDL-C	-0.0099	0.8640
TG	0.0292	0.6147
CRP	0.1813	**0.0016**

Sample numbers, correlation coefficients (Rs values) and P-values obtained using Spearman's correlation analysis are shown. [*]Subject data used are listed.[b]Dialysis period (yera) until blood collection, [c]Significant correlations ($P<0.05$) are indicated in bold font. BMI, body mass index; max-IMT, maximum intima-media thickness; CAVI,

cardio-ankle vascular index;, ABI, ankle brachial pressure index; HbA1c, glycated hemoglobin;W-PTH, whole parathyroid hormone; ARB, angiotensin II receptor blocker;ACE, angiotensin converting enzyme; PTA, prothrombin; Fe, iron; TSAT ratio,transferrin saturation ratio;Kt/V, standardized urea clearance; RBC, red blood cell number; HGB, hemoglobin; HCT, hematocrit; PLT, platelet number; TP, total protein; UN, urea nitrogen; CRE, creatinine;UA, uric acid; Na, sodium;K, potassium; Cl, chlorine; Ca, calcium; IP, inorganic phosphate; Mg, magnesium;AST, aspartate aminotransferase;ALT, alanine amino transferase; LDH, lactate dehydrogenase;γ-GTP, γ-glutamyl transpeptidase; ALP, alkaline phosphatase; tBil,total bilirubin; AMY, amylase; CK, creatinine kinase; T-CHO, total cholesterol; HDL-C, high-density lipoprotein cholesterol; LDL-C, low-density lipoprotein cholesterol;TG, triglycerides; CRP, C-reactive protein.
